# Supplementary material for: Are carbon emissions trading and green financial instruments synergistic? -Comprehensive quantitative research based on content analysis
Source: PLoS One. 2024 Mar 7;19(3):e0298601. doi: 10.1371/journal.pone.0298601 (PMC10919601; doi:10.1371/journal.pone.0298601)
Supplement: S1 Data — (ZIP) [file pone.0298601.s001.zip › Data/地级市Z_F工作报告.zip!/╣π╢1⁄2/│▒╓▌╩╨/└┤╫╘╣1⁄2╓┌║┼í╛┬φ┐╦╩2╛▌═°í┐_2016.docx]

各位代表：

现在，我代表市人民政府向大会作工作报告，请予审议，并请市政协委员和其他列席人员提出意见。

“十二五”时期的工作回顾

“十二五”时期，是我市发展史上很不平凡的五年。我们在省委省政府和市委的正确领导下，在市人大及其常委会和市政协的监督支持下，坚持以党的十八大和十八届三中、四中、五中全会精神为指导，深入学习贯彻习近平总书记系列重要讲话精神，全面落实省振兴粤东西北各项决策部署，谋划推进“一中心三片区”建设，统筹抓好稳增长、调结构、促改革、惠民生、防风险各项工作，“十二五”规划主要目标任务基本完成，为决胜“十三五”、决战2016年打下坚实基础。

“十二五”时期，是我市综合实力大幅提升的五年。我们坚持稳中求进，积极应对复杂严峻的经济形势，制订出台一系列扶持措施，着力促进经济持续发展，全市呈现经济增长较快、质量提升、后劲增强的良好态势。一是增长呈稳定性。2015年，全市生产总值910.1亿元，比上年增长8.3%，年均增长10.2%；一般公共预算收入47.2亿元，比上年增长14.4%，年均增长15.2%；税收总收入92.3亿元，比上年增长1.4%，年均增长8.6%；城乡居民人均可支配收入1.68万元，比上年增长10.3%，年均增长12.8%。二是增长呈协调性。区域发展能力提升，投资、出口、消费均衡增长。2015年，全市固定资产投资391.9亿元，年均增长19.7%，五年累计达到1383亿元，是“十一五”时期的1.94倍；出口总值27.6亿美元，年均增长3.4%；社会消费品零售总额443.1亿元，年均增长13.4%。三是增长呈持续性。基础建设对发展的支撑能力显著增强，五年累计完成投资341亿元。其中：交通建设完成投资107亿元，厦深铁路建成通车，一大批省道改造项目和潮州港亚太通用码头一期工程、潮州港进港公路建成，潮惠、宁莞、大潮高速潮州段动工建设，潮州大桥、韩江东西溪大桥等项目加快推进，新改建县乡公路190公里、通村公路1245公里，立体化交通路网逐步形成；电网建设完成投资26.8亿元，新改建了一批电网重点工程，供电保障能力有效提升；信息化建设完成投资48亿元，信息网络体系更加完善；水利建设完成投资15亿元，沟尾溪、内洋南总干、河内湖三大涝区整治工程扎实推进，引韩济枫应急工程及一批农村水系连通工程完工投用。

“十二五”时期，是我市产业结构调整优化的五年。我们坚持创新引领，加快产业转型升级，三大产业结构调整优化为

7.3:53.6:39.1。一是特色农业加快发展。现代农业园区、农业标准化示范基地及“菜篮子”基地建设扎实推进，农业龙头企业和流通组织示范带动作用持续增强，粮食种植面积保持稳定，单丛茶、铁皮石斛、水产养殖等特色农业加快发展，凤凰单丛乌龙茶资源利用和品质提升关键技术及产业化项目获得省科技奖一等奖。二是工业发展水平提升。陶瓷、服装等传统产业加快发展，电子、信息等新兴产业逐步形成，临港产业规模不断壮大，新增上市公司5家，新认定为高新技术企业37家。创新驱动及品牌标准战略有效实施，构建了中山－潮州“市、区、镇、企”四级创新联盟，建成一批质检机构、研发中心、专业镇，专利申请量和授权量均居全省第10位，我市被认定为国家知识产权试点城市。2015年，全市规模以上工业增加值361.2亿元，增长7.8%，年均增长16.2%。三是第三产业不断壮大。全市旅游规划启动修编，古城文化旅游区建设成效明显，引客入潮效果良好，2015年全市接待海内外游客921万人次，实现旅游收入141.6亿元。市跨境电子商务协会成立运营，与阿里巴巴、京东商城等合作平台有效搭建，饶平成为国家电子商务进农村综合示范县。商贸物流、金融服务等发展水平不断提升，2015年全市金融机构存款余额1076亿元、贷款余额369亿元，分别比年初增长7.2%、3.3%，年均增长10.5%和10.9%。

“十二五”时期，是我市全面深化改革开放的五年。我们坚持改革开放，以深化改革突破体制束缚，以扩大开放增创发展优势。一是重点改革稳步开展。财税、金融、投融资体制改革不断深化，旅游、环保、燃气等领域改革扎实推进，中小学招生考试、公共交通体制、公务用车制度等改革有效实施。土地承包经营权确权登记、“三资”交易平台建设、涉农矛盾化解等农村综合改革积极开展，集体林权制度改革全面完成。商事制度改革有效开展，营商环境不断优化，市场活力持续迸发。二是平台建设扎实推进。闽粤经济合作区核心区加紧建设，已动工项目17个。凤泉湖高新区建设进展顺利，引进项目65个、总投资140多亿元，动工项目41个。潮州新区总体规划及各类专项规划编制完成，粤东西北股权发展基金落地实施，韩东新城征地拆迁工作有效推进，51个重点项目布点实施。潮安高铁新城规划建设加快，东山湖现代产业园发展势头良好。三是开放格局更加全面。区域交流合作有新突破，与厦门市和美国旧金山市等建立友城关系，融入珠三角、海西经济区步伐加快，对接港澳能力增强，参与21世纪海上丝绸之路和中国－东盟自贸区升级版建设积极开展，驻泰国经贸代表处正式挂牌运作。中山－潮州对口帮扶工作扎实有效，汕潮揭同城化稳步推进。

“十二五”时期，是我市促进城乡协调发展的五年。我们坚持统筹兼顾，促进城乡统筹发展、区域协调发展、人与自然和谐发展。一是发展格局进一步优化。“一中心三片区”建设扎实推进，“八网＋产业”规划建设启动实施。部分县区行政区划调整顺利完成，潮安实现撤县设区，市区面积从152.5平方公里扩大至1414平方公里。二是生态建设进一步加强。新一轮绿化潮州大行动深入开展，海洋生态环境得到较好保护，矿产资源管理工作不断强化，违法违规用地受到严厉打击。全市森林覆盖率达到62.65%，建成绿道31.4公里。节能减排措施全面落实，重点污染物防治力度加大，农村生活垃圾“一镇一站、一村一点”处理设施全面完成。三是宜居环境进一步提升。“三治”行动深入实施，宜居城乡建设扎实开展，省级新农村示范片建设有效推进，创建省级宜居社区8个、示范村8个、示范镇3个。

“十二五”时期，是我市社会事业明显进步的五年。我们坚持民生优先，加强以改善民生为重点的社会建设，让人民群众更加普遍、更加充分共享改革发展成果。一是民生福祉得到改善。积极就业政策有效实施，城镇新增就业、培训转移农村劳动力均超额完成年度任务。社会保障体系不断完善，城乡居民养老保险和医疗保险制度实现全覆盖，社会保险待遇大幅度提高。慈善福利事业有效开展，底线民生保障有力，扶贫开发任务全面完成。住房解困工作扎实推进，农村贫困户危房改造14723户，城镇各类保障性住房建设7489套。二是社会事业全面发展。全社会尊师重学氛围浓烈，教育创强创均工作成效明显，教育强镇、公办义务教育标准化学校建设覆盖率100%，各类教育均衡发展。公共文化服务体系逐步健全，传统文化艺术得到传承弘扬，拥有国家级非遗项目15项、传承人16人、传承基地2个、民间文化艺术之乡4个。医药卫生体制改革持续深化，公共卫生服务体系不断完善。计生政策有效落实，人口出生率、自然增长率均控制在省下达年度指标之内。三是社会大局保持稳定。“平安潮州”建设深入推进，社会治安防控体系不断完善，各类违法犯罪活动受到严厉打击。群体性事件得到妥善处置，各类社会矛盾纠纷有效化解。应急管理机制逐步健全，突发事件处置能力不断增强。安全生产形势保持稳定，产品质量和食品药品安全水平稳步提升，连续五年没有发生重特大安全事故和食品药品安全事件。与此同时，人事、审计、统计、法制、贸促、物价、粮食、打私、打假、人防、气象、防震、地方志、档案、对台、拥军优抚、民兵预备役、老龄、民族宗教、妇女儿童等工作取得新成绩。

在经济社会取得新发展的同时，法治政府建设继续加强。依法行政深入推进，自觉接受人大、政协监督，累计办理人大代表建议199件、政协提案402件。第五轮行政审批制度改革有效推进，市级机构改革全面完成，网上办事大厅拓展完善。党风廉政建设责任制全面落实，政务公开、行政监察、审计监督、网络问政、纠风工作力度加大，一批违法违纪案件受到严肃查处。

刚刚过去的2015年，市政府全面贯彻落实省委省政府和市委各项决策部署，围绕备战“十三五”的各项谋划，统筹推进各项工作，取得明显成效。一是谋划潮州发展新格局。继续紧抓交通扩网提速、园区扩能增效、城市扩容提质“三大抓手”，厦深高铁潮汕站规划升级为粤东区域中心枢纽站；编制全域规划，谋划“八网+产业”发展，启动闽粤经济合作区和高铁新城建设，推进韩东新城和凤泉湖高新区发展，“一中心三片区”发展格局初步形成。二是增强加快发展新动力。开展八场次境内外招商引资活动，加强与大唐集团、省交通集团等央企、省国企合作，积极推进投融资平台建设，获得省七大银行综合授信708亿元，成功举办潮州婚纱晚礼服时尚周，有效激发市场主体活力。三是创造社会建设新成果。深入推进以抓“三治”、清“三资”、治“三乱”的“三大整治”，开展“文明镇街、文明村居”创建活动，抓好凤凰洲、瓷泥厂、交通秩序等综合整治，推动“平安潮州”建设取得新突破，成为粤东最安全城市；设立潮州公益文化基金、韩愈教育发展基金、绿色生态发展公益基金，成功举办第六届粤东侨博会，创造了抗击登革热疫情的地方范例，申获省旅游综合改革示范市和省新型城镇化综合试点市。四是提升政府工作新水平。启动基层工作推进月活动，建立政府顾问制度，举办政府顾问决策咨询年会，下放市级审批权限193项，争取并获得人大立法权，全面落实省委巡视组反馈意见整改工作，推动政府工作务实有效开展。

各位代表，“十二五”期间，特别是2015年取得的成绩来之不易。这些成绩的取得，是省委、省政府和市委正确领导的结果，是市人大、市政协有效监督和大力支持，以及驻潮部队、武警官兵、驻潮部省属单位和社会各界鼎力相助的结果，更是全市广大干部群众辛勤努力、奋力拼搏的结果。在此，我代表市人民政府表示衷心感谢并致以崇高敬意!

各位代表，过去五年，我们不仅在应对挑战中取得了明显的发展成果，而且积累了在复杂环境中加快发展的宝贵经验。一是必须坚持发展第一要务，把上级精神和潮州实际紧密结合起来，找准着力点和突破口，创造性地开展工作；二是必须抢抓发展机遇，创新发展理念和发展模式，加快转变经济发展方式，不断增强发展的全面性、协调性和可持续性；三是必须具备全球视野和辩证思维，善于从战略上谋划潮州发展，对事关长远的重大问题进行系统设计和周密部署；四是必须依靠改革创新破解发展难题，加强重点领域建设，改善发展环境，增强发展后劲；五是必须坚持以人为本，把保障和改善民生作为发展的根本目的，充分发挥各方面的积极性和创造性，形成加快发展的强大合力。

同时，我们也清醒地认识到，我市经济社会发展中还存在不少矛盾和问题。一是经济总量不够大，市场主体不够多，创新能力不够强，发展质量效益不高。二是农业现代化步伐偏慢，工业集约集聚程度不高，服务业发展相对滞后，产业结构不够优化。三是资源环境约束日益明显，“两违”整治任务艰巨，城乡发展环境仍亟待改善。四是关系群众切身利益的问题仍然较多，人民生活水平提高不够快，公共服务供给能力依然不足。五是政府职能转变不到位，少数政府工作人员存在形式主义、官僚主义、担当不够和消极腐败现象，依法行政能力仍需进一步增强。

“十三五”时期的目标任务

“十三五”时期，是我市加快“一中心三片区”建设、实现凤凰展翅腾飞的重要时期，既面临机遇，也存在挑战，但总体上机遇大于挑战。我们必须认清新形势、适应新变化，立足新常态、完善新思路，抓住新机遇、推动新发展。综合考虑我市发展趋势和各种因素，预期发展目标为：生产总值年均增长11%以上，到2020年达到1700亿元以上；人均生产总值年均增长10.5%以上，到2020年达到1万美元；一般公共预算收入年均增长14%以上，力争到2020年达到100亿元；固定资产投资力度加大，年均增长25%左右；城乡发展差距缩小，城镇化水平提高到66%以上。

“十三五”时期，政府工作的指导思想是：全面贯彻党的十八大和十八届三中、四中、五中全会精神，以邓小平理论、“三个代表”重要思想、科学发展观为指导，深入学习贯彻习近平总书记系列重要讲话精神，围绕“五位一体”总体布局和“四个全面”战略布局，围绕省委省政府振兴粤东西北战略部署，围绕“一中心三片区”战略定位，牢固树立“创新、协调、绿色、开放、共享”发展理念，坚持全面深化改革，强化创新驱动引领，推进城乡协调发展，建设绿色生态家园，不断扩大对外开放，切实增进民生福祉，确保全面建成小康社会。

“十三五”时期，我们将突出重点抓好以下工作：

（一）坚持创新发展，激发经济原生动力。深化经济体制改革，创新行政管理体制，破解经济发展中的体制性、结构性矛盾，使市场在资源配置中起决定性作用。实施创新驱动战略，深化科技体制改革，推动创新链与产业链、资金链深度融合，加大人才培育引进力度，加强新型智库建设，促进大众创业、万众创新，建设创新型潮州。

（二）坚持协调发展，优化区域空间布局。深入实施全域规划，推进“八网+产业”建设，着力打造“一中心三片区”，优化区域经济空间布局。注重产城融合发展，推进省新型城镇化综合试点市建设，加快“三农”现代化步伐。深入贯彻“中国制造2025”，推动特色产业转型升级，培育战略性新兴产业，大力发展现代服务业，发展提升文化产业，积极孕育新业态和新商业模式，构建具有区域特色的现代产业体系。

（三）坚持绿色发展，建设潮人生态家园。深化生态文明体制改革，推进绿色发展示范区建设，培育保护生态系统，抓好节能减排工作，促进资源节约集约利用，倡导绿色低碳生活，推动“三治”工作取得更大成效，积极构建资源节约型和环境友好型社会，全力建设天蓝、地绿、水净的美好家园。

（四）坚持开放发展，增强经济发展后劲。全面贯彻“一带一路”战略，综合运用国际国内两个市场、两种资源，统筹对内对外开放，抓好“海丝”、闽粤、中潮“三大合作”，坚持“引进来”和“走出去”并重，扩大港口辐射带动作用，增创开放型经济发展新优势，把潮州打造成具有国内示范性、国际影响力的“海丝”节点城市，塑造“海丝文化重镇、潮人精神家园”城市形象。

（五）坚持共享发展，增创社会民生福祉。强化政府公共服务职能，促进基本公共服务均等化，努力保障底线民生，完善社会保障体系，加快发展教育事业，促进文化传承发展，提高人民健康水平，推进平安潮州建设，增强人民群众幸福感。积极践行社会主义核心价值观，传承“创业、精致、感恩、包容”优秀文化特质，打造国际文化品牌，提升文化软实力。

（六）坚持依法行政，打造有力有为政府。坚持依法治市、依法执政、依法行政共同推进，坚持法治市场、法治政府、法治社会一体建设，全面建设法治潮州，实现科学立法、严格执法、全民守法，促进治理体系和治理能力现代化。改革创新行政服务体系，加快延伸网上办事大厅，全面清理行政职权，推进行政审批标准化，建设“阳光政府”，促进行政权力高效规范运行。

2016年主要工作

今年是决胜“十三五”、实现全面小康的开局之年，是美好蓝图落实落地之年。当前，国内外经济环境依然复杂严峻，但新的经济增长动力正孕育形成，做好今年政府工作意义重大。根据“十三五”发展规划，并综合考虑各种因素，今年全市经济社会发展预期目标是：生产总值增长10.5%以上，一般公共预算收入增长13.5%以上，固定资产投资增长26%，出口总值增长5%，社会消费品零售总额增长12%，居民消费价格指数涨幅控制在3%以内，城镇登记失业率控制在3.5%以下，城乡居民收入增长与经济增长基本同步，节能减排降碳约束性指标完成省下达的年度计划。

为实现上述目标，今年政府工作的指导思想是：牢固树立创新、协调、绿色、开放、共享的发展理念，围绕中央、省委省政府和市委十三届六次全会的各项部署，以后发赶超为目标导向，以落地落实为重要抓手，以有力有为政府为支撑，大力弘扬创业拼搏的“红头船”精神和追求精致的“一根牛绳”精神，深入践行“三严三实”，适应和引领新常态，突出重点项目带动，建好重大发展平台，扎实推进“三大抓手”，加快实施“八网”建设，促进产业创新发展，大力推进改革开放，不断优化发展环境，切实增进民生福祉，奋力建设“一中心三片区”，全力决战2016年，为决胜“十三五”、实现全面小康开好局、起好步。

（一）实施项目攻坚行动

坚持项目为王、动工为先，突出项目带动引领，加强重点项目建设，增强经济发展后劲。

推进八网重大项目。深入实施三年重点基础设施建设专项行动计划，全力抓好年度投资163.6亿元的78个重点项目，突出推进燃油燃气网、供电网、互联网、水网、综合交通网、绿道网、商贸物流网、旅游网“八网”和产业重大项目建设，重点加强管道燃气供应“一张网”、华丰和华瀛等液化天然气储备站、邹鲁等输变电和新建配网工程、信息基础设施、粤东（潮州）灌区改造工程、一江两岸文化生态提升工程、古城特色旅游区、广梅汕增建二线和潮惠、宁莞、大潮高速公路及饶平火车站疏站北路等项目建设，改造提升一批国省道，动工建设潮汕环线高速、梅汕客运专线潮州段和潮汕站动车运用所，做好潮州港疏港铁路前期工作。

促进项目开工建设。继续推行项目保姆式服务，落实“一线工作法”，开通审批绿色通道，缩短项目审批时限。建立项目落地促建机制，加强签约项目跟踪协调，做大项目储备规模。狠抓项目前期工作，倒排时间进度表，逐个环节明确时限，夯实开工建设基础，确保每季度首月均有一批项目动工建设。

完善项目推进机制。实施重点项目领导挂钩督导制度，建立“年初建账、季度对账、半年查账、年底结账”台账式管理机制，制定项目建设路线图，推进项目全程跟踪督办，及时破解审批、融资、征地、拆迁等要素制约，力促项目顺利推进。加强在建项目服务，扩大资金、人力、设备投入，形成更多投资量。加强银政、银企合作，探索金融机构直接融资及PPP合作模式，引进各类资本参与基础设施和公用事业建设与经营。

（二）加快重大平台建设

围绕打造“一中心三片区”目标，突出四大发展平台建设，加强土地征收储备和招商引资工作，形成新的经济增长点。

推进韩东新城建设。贯彻“拓东先拓中、重点在南部、北部自然成”建设思路，推进新城51个重点项目建设。启动潮州东大道、东山路升级改造项目，基本完成潮州大桥、韩江东西溪大桥建设任务。加快东方国际茶都、潮州恒大城等项目建设，扎实抓好潮商城、华都博览城等项目前期工作，完善新城商务办公、教育医疗、文体娱乐、高品质居住等公共服务功能。

推进高铁新城建设。加强高铁新城整体形象设计宣传，启动厦深高铁潮汕站中心站场详规和沙溪镇中心城镇规划编制，推进厦深高铁潮汕站扩建工程，加快商贸物流中心项目建设。完善东山湖现代产业园基础设施，抓好梅林湖旅游开发商住区征地拆迁工作，引进培育更多产业项目。

推进闽粤经济合作区建设。全面加强与漳州市的沟通对接，抓好合作区规划实施方案及各类专项规划编制，共同争取获得国家更多政策支持。推进投融资平台创建，加快文胜围南片一期土地平整工程和潮州港扩建货运码头工程、口岸综合服务中心等项目建设。开工建设潮州港公用航道一期工程，推动中塞环保建筑材料综合利用、液化天然气接收码头储配及管线等项目尽快建设。强化产业项目支撑，争取有更多项目落地，形成产业集聚效应。

推进凤泉湖高新区建设。做好高新区扩区更名申报工作，推进高新区“镇园合一”产业新城规划。完善园区基础设施配套，加快推进污水处理厂、中山大道建设。加快已获地企业促建工作，推动湖北荣信投资管理有限公司、上海明园集团新型金属质感材料等重大项目建设，力争一批项目投产。创新投融资模式，积极与国内大型企业洽谈合作开发园区。全面加强与中山市对口合作，组织开展各类招商推介活动，力争在招商引资上取得新突破。

（三）推进产业创新发展

围绕主导产业，坚持创新驱动，打造产业集群，完善产业体系，促进实业兴市。

改造提升传统产业。贯彻中国制造2025，实施创新驱动发展战略，推进产业提升三年行动计划，优化升级传统特色产业，培育发展高新技术产业。深入开展质量强市行动，加强科技企业孵化器和企业研发机构建设，大力开展技术改造和机器换人，加快生产工艺革新和技术装备现代化，促进新型工业化与信息化深度融合。推动各类资源要素向优势企业聚集，引导优势企业规改股、股上市，培育大型骨干企业。推进国家新型工业化产业示范基地建设，大力发展新材料、节能环保、电子机电、生物制药等新兴产业，以优质增量稀释低端存量。

培育壮大房地产业。启动房地产业十年发展规划编制，优化房地产业发展方向，规范开发秩序，提升监管服务水平。探索完善征地补偿安置机制，推进以满足新市民为出发点的住房制度改革，扩大有效需求，稳定房地产市场。加快韩东片区、潮州碧桂园等“三旧”改造项目建设，推动县域房地产业发展壮大。

大力发展第三产业。深入实施“互联网+”行动计划，加快电子商务产业园建设，积极打造“线上”潮州特色产品专馆，推进“一村一品”农产品电商发展。探索设立“创业孵化基地”、“众创空间”等新型创新创业服务平台，加快建设财富中心、恒德国际大厦等城市中央商务区，完善城市商业功能配套。争取中国国际时装周婚纱晚礼服专场常年落户潮州，带动和激发时尚消费产业发展。推进国家旅游综合改革试点市创建，抓好笔架山潮州窑、广济桥、柘林港申报世界文化遗产首期综合工作，加快星级酒店、古城文化旅游设施等配套建设，新改建旅游厕所83处，全面提升旅游产业发展承载力。

加快建设现代农业。做好第三次全国农业普查工作。编制绿色发展示范区发展规划，推动水果和有机蔬菜生产带、农业高新产业生产带、单丛茶特色产业带、畜禽发展示范点建设，提升特色农业发展水平。加快发展农产品精深加工业和流通业，大力发展休闲观光农业、农家乐、乡村游等新业态。编制现代渔港建设总体规划，促进三百门国家一级渔港项目竣工投用，发展壮大深水网箱产业园。落实强农惠农政策，推进高标准农田建设，推广应用农业新技术，稳定粮食和主要农产品供给，促进农民增收。

（四）提升改革开放水平

全面深化改革，进一步扩大开放，切实用好内外两个市场、两种资源，着力打造转型发展新引擎。

激发市场主体活力。加大改革攻坚力度，推进供给侧结构性改革，着力促进去产能、去库存、去杠杆、降成本、补短板，提高全要素生产率。推动商事制度改革各项措施落地，积极推进“三证合一”、“一照一码”登记制度，发展壮大市场主体，做好“增量”文章。申报设立中国潮州（陶瓷）知识产权快速维权中心，维护创新主体权益。推进金融改革创新，以服务实体经济为导向，完善多层次资本市场。深化国企国资改革，大力促进民营经济发展。完成大学生创业孵化基地暨人才服务管理平台建设，修订完善高层次人才引进办法，促进大众创业、万众创新。

培育出口消费热点。推动外贸流程再造，发展壮大供应链龙头企业和外贸流通型企业，探索发展旅游购物出口贸易，培育商业新模式。组织企业参加各类展销会，推动企业在外设立研发中心和商品展示中心，拓宽营销渠道，开拓多元化市场。加快培育进口商品交易中心，推进潮州港三百门码头进口肉类指定口岸建设，扩大食品类和能源资源进口。组织开展各类展销、促销活动，完善商贸物流网，深入挖掘消费潜力，打造新兴消费热点。

扩大招商引资成效。以园区为依托，连结周边专业镇，开展重点产业招商，实行省市联合定向招商，促进优质项目向园区集聚发展。积极参与“跨国公司总部行”活动，争取省招商引资专项资金扶持，支持企业开展境外品牌、技术和产能并购，推动世界500强企业和大型跨国企业在潮投资发展，提升利用外资水平。

促进区域交流合作。积极参与“粤企丝路万里行计划”。做好友城拓展工作，优化对外交往布局。依托闽粤经济合作区，扩大与福建、台湾的交流合作。强化与中山市的联系，促进对口帮扶工作深入开展，争取更多项目落地。加强与汕头、揭阳的对接，共同推进临港空铁经济合作区建设，促进汕潮揭同城化发展。

（五）构筑绿色生态环境

深入开展“抓三治、清三资、治三乱”工作，切实加强环境保护，全面提升生态文明建设水平，创建粤东最干净城市。

加大“三治”力度。深入开展韩江、黄冈河整治，切实保护好饮用水源。强化枫江流域治理，努力改善枫江水质。严格污染源头管控，确保达标排放。加强大气污染防治，加大黄标车淘汰力度，在市区部分道路实施喷雾降尘，提高空气质量。继续开展“文明镇街、文明村居”创建活动，扎实抓好城乡环境卫生整治工作。推进潮安垃圾焚烧发电厂和饶平大湖山生活垃圾卫生填埋场建设，完善镇、村生活垃圾处理体系。

保护自然生态。加强耕地保护，加大闲置土地清理处置力度，大力整治违法违规用地，修编土地利用总体规划，强化用地服务保障，促进节约集约用地。开展矿产资源规划修编，抓好地质灾害防治工作，加强桑浦山环境综合治理。规范河道采砂管理秩序，严厉打击违法采砂行为。完成柘林湾海洋生态修复，加强西澳岛生态保护，推进美丽海湾规划建设。打造绿色生态屏障，建好5个万亩特色基地、2个特色公园，新建防护林3.2万亩、森林公园9个、湿地公园1个，新增绿化美化乡村118个。

促进节能减排。严格落实节能责任制，推进电机能效提升，加强“三能”体系建设。抓好循环经济工业园和省市共建循环经济示范园等创建申报工作，推动清洁生产。抓好5个污水处理厂建设，加快完善相关配套管网，全面推进规模化畜禽养殖场污染整治，提升污染减排能力。

（六）建设现代宜居城市

加强城市建设管理，推动城市硬件设施和软实力双提升、人与城市共发展。

加快新型城镇化进程。扎实抓好省新型城镇化综合试点市建设工作，加强城镇体系规划编制，推进省级新农村示范片和名镇名村示范村建设。完成内洋南总干和沟尾溪涝区整治工程，推进内洋西总干、河内湖和归湖、江东涝区整治。提升农村饮水安全水平，全面推进村村通自来水工程。加强农村道路建设，新改建县乡公路20公里。深化农村改革，完善乡村治理，化解涉农矛盾，铺开县、镇、村（社区）三级公共服务平台建设。完成农村土地承包经营权确权登记颁证，推进农村集体建设用地流转试点工作。完善数字潮州地理信息公共平台。

提升城市管理水平。完成潮州市城市总体规划纲要、市中心城区生态控制线规划、“三旧”改造规划修编和“十三五”近期建设规划等编制工作。尊重城市发展规律，统筹城市空间、人口、规模、产业等要素配置，积极防治“城市病”。推进枫溪广场、绿榕北路、北园路和西荣路改造，动工建设外环西路、金塘路、北站二路、兴工北路等道路工程，逐步改善城市交通组织水平。建成开放凤城公园，加快凤凰洲环岛绿道建设，提升道路绿化层次感和舒适度。推进城市管理体制改革，成立市城市管理执法支队，加大“六乱”整治力度，营造良好城市环境。

彰显名城文化魅力。建设“城市规划展示馆”，打造城市规划展示窗口。加强古城文化旅游重点项目建设，逐步推进古城区街巷修缮改造，加快义安路沿街改造提升，启动饶宗颐旧居修缮工作，建设潮州美食城。加强城市特色风貌设计，推动非物质文化遗产传承发展，完善历史建筑保护制度，打造个性化城市，焕发古城新魅力。发挥海外潮人纽带作用，不断扩大潮州文化影响力，着力塑造“海丝文化重镇、潮人精神家园”城市形象。

（七）优化提升公共服务

坚持守住底线、突出重点、完善制度，加大财政投入力度，推动民生工作再上新台阶。

加快发展社会事业。深入开展尊师重学活动，启动创建教育现代化先进市，全面完成教育创强工作。完善各学阶各类型学生资助政策体系，逐步提高覆盖面和资助标准，做好县域内义务教育阶段教师资源统筹配置。大力实施文化惠民工程，加快推进公共文化服务设施建设。支持开展群众体育运动，积极发展校园足球。继续深化医药卫生体制改革，启动市中心医院新院建设，引导优质医疗资源下沉，促进基本公共卫生服务均等化。深化爱国卫生运动，加强突发公共卫生事件应急处置，强化重大疾病防控。实施“全面两孩”政策，做好新旧政策衔接工作，促进人口均衡发展。继续做好民族宗教、广播电视、新闻出版、拥军优抚、国防动员、预备役、对台、人防、气象、防震、统计、物价、档案、地方志、老龄、妇女儿童、残疾人、红十字等工作，促进各项事业全面发展。

完善社会保障体系。深入实施更加积极的就业政策，新增城镇就业2万人、失业人员再就业4000人。落实创业培训补贴，完善基层人力资源和社会保障公共服务网络，推动创业带动就业。推进社保扩面征缴，健全社会保障机制，开展机关事业单位养老保险制度改革，全面实施全民参保登记计划，拓展“电子社保”服务范围，努力实现社会保障“一卡通”。全面推进精准扶贫、精准脱贫，实施基础设施建设、产业化帮扶等扶贫工程，加快形成全社会参与的扶贫大格局。加大住房解困力度，完成4591户农村低收入住房困难户住房改造任务，实现公共租赁住房保障450户。

办好十项民生实事。围绕群众需求，加大资金投入，全力办好十项民生实事。一是巩固提升底线民生保障水平；二是增强医疗保障能力；三是强化低收入住房困难群众住房保障；四是促进教育资源公平均衡配置；五是改善农村生产生活条件；六是促进就业创业工作；七是加强污染治理和生态建设；八是强化公共安全保障；九是抓好防灾减灾工作；十是加大低收入弱势群体帮扶解困力度。

推进社会治理创新。加强社会治安视频监控系统建设，完善立体化社会治安防控体系，严打各类违法犯罪。全面落实各项防范措施，坚决防范和遏制重特大生产安全事故发生。推动阳光信访和网上信访，加强各类矛盾纠纷排查化解，提升应急管理能力，营造和谐稳定的社会环境。大力推进食品安全示范区和溯源体系建设，强化食品药品网格化管理，确保人民群众饮食用药安全。

（八）建设有力有为政府

认真落实省委巡视组反馈意见整改工作，坚持依法行政，改进行政服务，打造法治廉洁政府，全面提升政府整体工作水平。

坚持目标导向。围绕年度目标任务，明确各阶段工作，倒排时间进度表，落实工作责任人，强力推进工作落实。认真践行“三严三实”，做到敢于担当、勇于作为、善于成事，聚焦发展瓶颈，解决实际问题，强化工作保障，对确定的目标、制定的政策、部署的任务，一抓到底、务求实效。

坚持依法行政。全面推进法治政府建设，完善政府顾问和法律顾问制度，严格执行重大行政决策程序。规范行政执法工作，推进行政复议委员会试点，强化层级监督，坚决纠正违法或不当行政行为。认真执行人大及其常委会各项决议决定，及时办理人大代表建议和政协提案，自觉接受人大、政协和社会监督。

坚持高效服务。健全基本公共服务体系，努力为人民群众提供更多更好的公共产品和服务，为各类市场主体创造公平竞争的市场环境。深化行政审批制度改革，完善行政服务管理体系，加快行政服务管理中心改革重组，推行“一门式”、“一网式”政务改革，探索开展审批权“三集中、一分离”工作。完成大交通管理体制改革试点，筹建市政府投资项目代建管理局，研究推动旧城区街道撤并工作，提高政府工作管理运行效能。

坚持改进作风。严守政治纪律和政治规矩，严格执行中央八项规定，持之以恒纠正“四风”。强化宗旨意识，密切联系群众，做到察实情、办实事、求实效，着力解决群众反映强烈的突出问题。厉行勤俭节约，反对奢侈浪费，建设节俭政府。

坚持廉洁从政。严格落实党风廉政建设责任制，认真履行“一岗双责”，推进廉洁政府建设，坚持用制度管权管事管人。深入实施“阳光政务”，全面深化政务公开。抓好政府系统党风廉政建设和公务员队伍建设，促进干部清正、政府清廉、政治清明。

各位代表！新起点肩负新重任，新形势要有新作风。让我们紧密团结在以习近平同志为总书记的党中央周围，在市委的坚强领导下，解放思想、锐意创新，凝心聚力、真抓实干，敢于担当、有力有为，决战2016年，决胜“十三五”，为建设“一中心三片区”、实现潮州后发赶超而努力奋斗！

附件：

2016年市十件民生实事

一、巩固提升底线民生保障水平。城乡居保基础养老金标准提高到每月110元；城镇、农村低保补助补差水平分别提高到每月418元和190元；农村五保供养标准不低于当地上年度农村居民人均可支配收入的60%；孤儿集中供养水平提高到每月1340元，分散供养水平提高到每月820元；城乡医疗救助人均补助标准提高到每年2178元，政策范围内住院医疗救助比例提高到70%以上；残疾人生活津贴每年1200元，重残护理补贴每年1800元。

二、增强医疗保障能力。城乡居民医疗保险补助标准提高到年人均不低于410元；城乡居民基本医疗保险年度支付限额A档提高到24万元、B档提高到30万元；城乡居民大病保险报销比例提高5-10个百分点，年度支付限额相应提高；完成乡镇卫生院业务用房改扩建年度目标任务。

三、强化低收入住房困难群众住房保障。完成4591户农村低收入住房困难户住房改造任务；鼓励公租房保障货币化，通过实物保障或货币保障，实现公共租赁住房保障450户。

四、促进教育资源公平均衡配置。市属中职学校（含技工学校）的免学费补助标准每生每年提高到3500元；农村寄宿制学校生均住宿费补助标准提高到每年200元；学前教育困难家庭幼儿资助标准提高到每年1000元；把山区和农村边远地区学校教师补贴政策实施对象扩大到公办普通高中和公办幼儿园，补助标准提高到人均不低于每月800元。

五、改善农村生产生活条件。提升农村饮水安全水平，全面推进村村通自来水工程；推进普惠金融“村村通”试点工作，乡村金融服务站和助农取款点实现行政村全覆盖；新改建县乡公路20公里；推进县、镇、村（社区）三级公共服务平台建设。

六、促进就业创业工作。实施更加积极的就业政策，新增城镇就业2万人、失业人员再就业4000人。落实创业培训补贴，完善基层人力资源和社会保障公共服务网络，推动创业带动就业。全面完成大学生创业孵化基地暨人才服务管理平台建设。

七、加强污染治理和生态建设。加快推进农村生活垃圾“一县一场”设施和5个污水处理厂及配套管网建设。建成开放凤城公园，新建防护林3.2万亩、森林公园9个、湿地公园1个，新增绿化美化乡村118个，新改建旅游厕83处。

八、强化公共安全保障。启动“电信诈骗防火墙”工程；全面配齐全市综合应急救援队伍器材装备；在全市选取部分条件成熟的农贸市场（含超市）开展食品安全快速检测工作。

九、抓好防灾减灾工作。完成内洋南总干和沟尾溪涝区整治工程，推进内洋西总干、河内湖和归湖、江东涝区整治。

十、加大低收入弱势群体帮扶解困力度。全面推进精准扶贫，实施基础设施建设、产业化帮扶等扶贫工程，引导形成全社会扶贫格局；用于社会福利事业的彩票公益金，50%以上的资金用于支持发展养老服务业；对因遭遇意外事件、突发重大疾病或其他特殊原因导致基本生活出现严重困难的家庭和个人给予临时救助。
